# Acclimatization of massive reef-building corals to consecutive heatwaves

**DOI:** 10.1098/rspb.2019.0235

**Published:** 2019-03-06

**Authors:** Thomas M. DeCarlo, Hugo B. Harrison, Laura Gajdzik, Diego Alaguarda, Riccardo Rodolfo-Metalpa, Juan D'Olivo, Gang Liu, Diana Patalwala, Malcolm T. McCulloch

**Affiliations:** 1 Australian Research Council Centre of Excellence for Coral Reef Studies, Oceans Graduate School, The University of Western Australia, Crawley, Western Australia 6009, Australia; 2 Centre for Microscopy, Characterisation and Analysis, The University of Western Australia, Crawley, Western Australia 6009, Australia; 3 Australian Research Council Centre of Excellence for Coral Reef Studies, James Cook University, Townsville, Queensland 4811, Australia; 4 School of Molecular and Life Sciences, TrEnD Laboratory, Curtin University, Western Australia 6102, Australia; 5 Mediterranean Institute of Oceanography, Aix-Marseille University, Campus de Luminy, Marseille 13009, France; 6 ENTROPIE IRD—Université de La Réunion—CNRS, Nouméa 98848, Nouvelle-Calédonie, France; 7 US National Oceanic and Atmospheric Administration, Coral Reef Watch, College Park, MD 20740, USA; 8 Earth System Science Interdisciplinary Center/Cooperative Institute for Climate and Satellites-Maryland, University of Maryland, College Park, MD 20740, USA

**Keywords:** coral reefs, acclimatization, climate change, ocean warming, coral bleaching

## Abstract

Reef-building corals typically live close to the upper limits of their thermal tolerance and even small increases in summer water temperatures can lead to bleaching and mortality. Projections of coral reef futures based on forecasts of ocean temperatures indicate that by the end of this century, corals will experience their current thermal thresholds annually, which would lead to the widespread devastation of coral reef ecosystems. Here, we use skeletal cores of long-lived *Porites* corals collected from 14 reefs across the northern Great Barrier Reef, the Coral Sea, and New Caledonia to evaluate changes in their sensitivity to heat stress since 1815. High-density ‘stress bands’—indicative of past bleaching—first appear during a strong pre-industrial El Niño event in 1877 but become significantly more frequent in the late twentieth and early twenty-first centuries in accordance with rising temperatures from anthropogenic global warming. However, the proportion of cores with stress bands declines following successive bleaching events in the twenty-first century despite increasing exposure to heat stress. Our findings demonstrate an increase in the thermal tolerance of reef-building corals and offer a glimmer of hope that at least some coral species can acclimatize fast enough to keep pace with global warming.

## 1. Introduction

Since the beginning of the industrial era in the mid-nineteenth century, anthropogenic greenhouse gas emissions have driven an increase in tropical sea surface temperature (SST) of +0.7°C, with an even greater warming of +0.9°C in coral reef regions [1,2]. Global climate models project an additional +1°C warming by the end of this century even if drastic emissions reductions are implemented to avoid the +2 to +4°C warming likely to occur under a business-as-usual scenario [1]. Rising SST is devastating for reef-building corals because warm anomalies of even +1°C can cause them to lose the symbiotic algae that are of paramount importance to their survival. This phenomenon is termed ‘coral bleaching’ as normally colourful corals turn white after expelling their pigmented symbionts [3,4]. Regardless of which actions are taken to tackle climate change, the world is already locked in to a level of warming considered sufficient to trigger annual mass bleaching on more than 90% of coral reefs worldwide by the end of the century [5,6].

The dire projections of coral reef futures are rooted in the assumption that corals bleach when water temperature exceeds a threshold level, and critically, that this threshold remains constant over time [6]. Coral bleaching is expected to occur when degree heating weeks (DHW, °C-weeks), a metric of heat stress that incorporates both the duration and magnitude of warming above the maximum monthly mean (MMM) temperature climatology, exceeds a threshold between 2 and 4°C-weeks [7–9], with mortality occurring between 3 and 8°C-weeks [8,9]. As global warming pushes summertime temperatures beyond the MMM more frequently, coral bleaching is anticipated to become more common until mass bleaching events reach annual frequency later this century [5,6]. Therefore, a future only exists for most coral reef communities if the threshold DHW that triggers bleaching keeps pace with rising SST. Yet, with few exceptions [10–12], little is known about whether coral tolerance to heat stress has changed over time, and whether there is potential for it to increase in the future as the oceans continue to warm.

**Figure 1.**
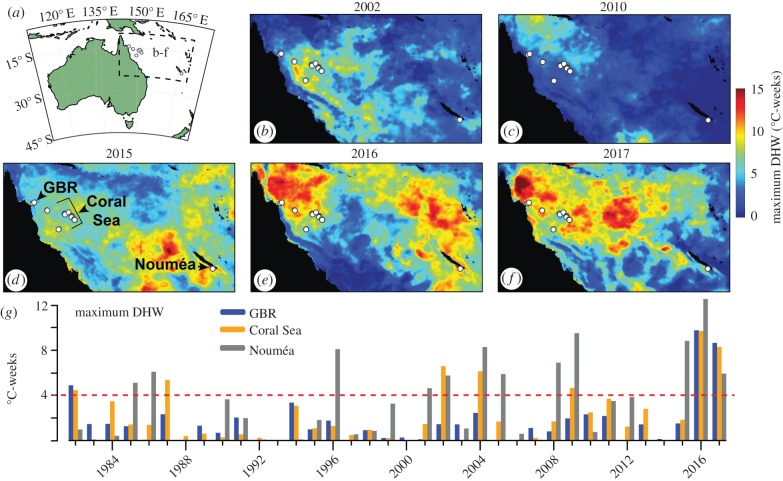
Heat stress on coral reefs in the twenty-first century. (*a*) Map of the Australia region, with dashed box indicating the GBR, Coral Sea, and Nouméa area. (*b–f*) Maps of maximum DHW at 5 km resolution per annum (colours) for key years with the highest DHW. (*g*) Annual maximum DHW for the northern GBR (blue), Coral Sea (orange), and Nouméa (grey). Dashed red line indicates the nominal 4°C-weeks bleaching threshold.

Here, we use the skeletons of long-lived *Porites* corals to evaluate their tolerance to rising ocean temperatures from 1815 to 2017 [2]. When *Porites* corals are bleached or otherwise physiologically perturbed by anomalous temperatures, they record their reaction within their skeletons as discrete high-density ‘stress bands’ that are visible in micro-computed tomography (μCT) scans. These historical stress events can be accurately dated using the annual density bands of the skeleton [13–19]. Together, this information enables (i) detection of stressful events—such as bouts of mass bleaching—that were not directly observed, and (ii) evaluation of the corals' sensitivity to heatwaves observed within recent decades. Our cores, collected in late 2017, span the northern Great Barrier Reef (GBR), northern Coral Sea atolls, and western New Caledonia (Nouméa) (figure 1*a*). Corals on these reefs were exposed to repeated warm SST anomalies in 2002, 2004, 2010, and 2015–2017 (figure 1; electronic supplementary material, figure S1), allowing us to assess their short-term (annual) responses to the increasing frequency of heat stress in the twenty-first century, in addition to their long-term (centennial) response to global warming since the early nineteenth century.

## 2. Methods

### (a) Climate data

We used the National Oceanic and Atmospheric Administration (NOAA) Coral Reef Watch (CRW) 5 km (v. 3.1) and 25 km SST and DHW products [9,20] to assess heat stress at our coral collection sites from 1982 to 2017. The 5 km dataset covered 1985–2017, and the 25 km dataset was used to extend the temporal coverage to include 1982–1984. We obtained the daily SST and DHW data for the single pixel covering each coring location, and for each year, we extracted the maximum DHW value. To make figure 1*g*, we averaged the maximum DHW per year for our northern GBR sites and our Coral Sea sites, separately. To make figure 1*b–f*, we downloaded the full daily 5 km CRW dataset for each year, and plotted (in colours) the maximum DHW at each pixel during each year. We validated these satellite SST data by comparing them to *in situ* temperature logger measurements wherever possible (electronic supplementary material, table S1 and figure S2) [21,22], which showed that the satellite-derived temperatures fall close to 1 : 1 lines with the logger data, with little evidence of local amplification effects [23].

### (b) Coral core collection

Coral skeletal cores were extracted from massive (i.e. dome- or hemispherical-shaped) *Porites* colonies using underwater pneumatic drills operated by divers. Cores from the northern GBR (*n* = 26) were collected between 1 and 10 m depth in September and October 2017 at Yonge Reef (*n* = 10; an outer barrier reef), MacGillivray Reef (*n* = 6; a fringing reef surrounding a sand cay), and Lizard Island (*n* = 10; sampling conducted on fringing reefs). In Nouméa, eight *Porites* colonies at 3 m depth were marked with submerged floats in March 2016, four that were bleached (those with names beginning with ‘B’) and four that were normally pigmented (those with names beginning with ‘Z’), and these eight colonies were then drilled in September 2017. Cores from the Coral Sea (*n* = 30) were sampled from colonies living between 1 and 20 m depth in November and December 2017 at Bougainville Reef (*n* = 9), Moore Reefs (*n* = 5), Diane Bank (*n* = 3), Willis Cays (*n* = 3), Magdalene Cays (*n* = 7), and Flinders Reef (*n* = 3). See electronic supplementary material, tables S2–S4 for additional core details. Northern GBR and Coral Sea cores were drilled with 5 cm diameter bits, and those from Nouméa with 2.5 cm diameter bits. Core holes were filled with cement plugs to prevent infestation by bioeroding organisms and to provide the corals with a hard surface to grow over during recovery. Each core was sonicated in an ultrasonic bath filled with deionized water for 15 min to remove particles, and then dried for at least 24 h in an oven at 50°C.

### (c) Micro-computed tomography scanning

All cores were scanned with a Skyscan 1176 (Bruker-microCT, Kontich, Belgium) to visualize stress bands, partial mortality scars, and annual density bands. Scan settings included voltage of 90 kV, current of 272 µA, a 0.11 mm Cu filter, 36 µm isotropic voxels, and step angles of either 0.7° or 1.1° over 180° rotations with no averaging. For Nouméa samples, we scanned the full core diameter with 350 ms exposures. For northern GBR and Coral Sea cores, we scanned the central 3 cm diameter of the cores with exposure times ranging from 500 to 1250 ms depending on density because longer exposures were required to achieve sufficient X-ray transmission for higher density cores. The scanner has a maximum scan length of 23 cm, so longer cores were scanned piece-wise and compiled to develop full chronologies (electronic supplementary material, figure S3).

### (d) Stress band analysis

All μCT scans were reconstructed into two-dimensional slices using Bruker Nrecon software with the following settings: beam hardening correction of 20%, ring artefact reduction of 12, smoothing of 3, and misalignment compensation adjusted each scanning day. Reconstructed images were converted to DICOM files in Hounsfield units (HU) with a range of −1500 to 500 HU. The reconstructed images were post-processed in MATLAB, averaging to 71 µm isotropic voxels to reduce file sizes, and then imported into Osirix software for visual analysis. We created digital slabs in the mean intensity projection with thicknesses between 2.5 and 3.0 mm to visualize both annual density banding and stress bands. The digital slabs were rotated and tilted within the three-dimensional image to locate the primary growth axis where banding patterns were most clearly visible. Annual density bands were counted backwards in time from the collection year of 2017 to establish a chronology for each core, following the well-established interpretation of annual density banding patterns [18,24]. Stress bands were identified following established methods [14,16,23] as either discrete, anomalous high-density bands, or partial mortality scars that may also include high-density stress bands slightly below the scar.

### (e) Dissepiment analysis

Dissepiments (thin horizontal skeletal elements accreted every lunar month [18]) were counted in the top 4 cm of cores following previously developed techniques [18]. Slabs were cut with a rock saw as close as possible to the primary growth axis, embedded in epoxy, and polished to a thin section. The sections were then imaged with an Aperio ScanScope XT digital slide scanner at 20× magnification. Dissepiments were traced on the digital images and counted back in time based on the number of full moons elapsed prior to the collection day [18]. Accurate analysis of dissepiment counts was only possible where the section was cut precisely along the growth axis [18], which precluded analysis of many sections and left us with a subset of 13 cores from the northern GBR with quality-controlled dissepiment counts. For two cores from Yonge Reef with dissepiment measurements reaching stress bands, the years of stress band formation interpreted from the μCT scans were confirmed by dissepiment counts. In other cases, the average dissepiment spacing was used to confirm the chronology interpreted from the μCT scans (see ‘Validation of chronologies').

### (f) Validation of chronologies

To validate our chronologies interpreted from μCT scanning, we compared our μCT-derived annual extension rates to those determined from dissepiment measurements (described above) and luminescent bands visible under ultraviolet (UV) light. An exceptionally bright luminescent band was visible in all the northern GBR cores between 7 and 10 cm from the top. By comparing the location of this bright luminescent band with μCT analysis of a core with clear density banding, we determined the bright luminescent band corresponds to late 2010 or early 2011 (electronic supplementary material, figure S4). We then divided the down-core depth of the bright luminescent band in all cores by 7 (approximately the number of years between its formation and the sample collection), and compared that to the average annual extension rate inferred from density banding. Additionally, we compared the average of the most recent 1–3 years of annual extension inferred from density bands to the average dissepiment spacing for these same corals multiplied by 12.4 (the number of lunar cycles per year). Both comparisons produced positive correlations close to 1 : 1 lines (electronic supplementary material, figure S5), thus generally confirming our interpretations of chronologies in the μCT scans.

### (g) Statistical analysis

We compiled our stress band identifications into a dataset filled with 0 for all years without any sign of a stress band and 1 for any year with a stress band and/or partial mortality scar. To test for temporal changes in the occurrence of stress bands, we fitted our stress band dataset to a generalized linear model with a binomial distribution and a logit link using the glmer function within the lme4 package in R. This test was performed with fixed effects of time and depth, reef location as a fixed factor, and random effects of individual cores.

We tested for temporal changes of sensitivity (proportion of cores with stress bands/maximum DHW per year) across the major stress events of the twenty-first century by fitting sensitivity per region (GBR, Coral Sea, Nouméa) to time with a simple linear model. For this test, we assessed the uncertainty of each sensitivity observation by conducting a Monte Carlo analysis and assuming that 10% of stress band assignments were false positives. The calculations were repeated 10^3^ times, and in each iteration, 10% of stress bands were randomly removed, enabling calculation of a standard deviation of sensitivity for each year.

The relationship between stress band occurrence and DHW during the twenty-first century bleaching events was tested by fitting with glmer the stress band dataset (0 and 1 s) to maximum annual DHW for each core during 2002, 2010, 2015, 2016, and 2017; including reef location and depth as fixed effects, and individual cores as random effects.

Additionally, we used odds ratios to evaluate whether cores with stress bands in a previous year were more likely to have another stress band in a latter year. Specifically, we calculated the odds ratios for the formation of 2010 stress bands compared to 2002 stress bands (i.e. were corals with 2002 stress bands more likely to have 2010 stress bands than corals without 2002 stress bands), and the same for 2010–2015, and 2015–2016. Odds ratios exceeding 1 indicated that prior stress banding had a positive effect on the likelihood of stress band formation in a latter year.

Summaries of statistical tests are provided in electronic supplementary material, table S5.

## 3. Results and discussion

**Figure 2.**
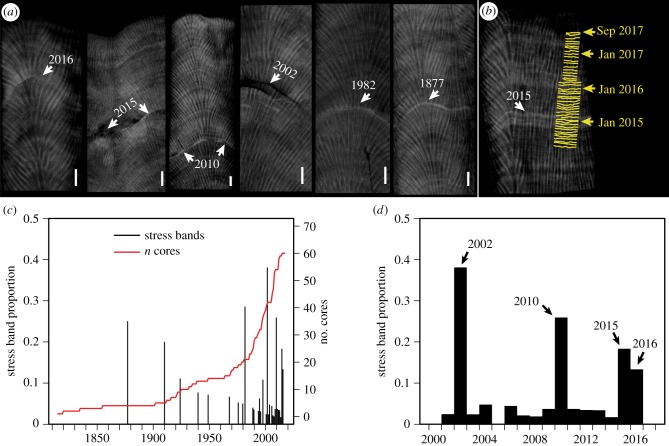
Coral skeletal core records of heat stress. (*a*) μCT scans (dark/light shading = low/high density) reveal discrete high-density stress bands and partial mortality scars preserved within the skeletons of long-lived *Porites* corals. Characteristic signatures of heat stress are shown for the years with the most stress bands recorded (2016, 2015, 2010, 2002, and 1982) and the oldest stress band in our dataset (1877). Scale bars are 5 mm. (*b*) Verification of the timing of a stress band as January–March 2015 is confirmed by monthly dissepiment counts (yellow) superimposed onto a μCT image of the same coral. (*c*) Stress band proportion (black bars) in all cores from 1815 to 2017 and (*d*) 2000–2017. The red line in (*c*) indicates the number of cores in the dataset per year.

The oldest stress band in our record corresponds to 1877 (figure 2), a year with a strong El Niño but prior to substantial industrial warming [2]. Following this pre-industrial heat stress event, the first widespread occurrence of stress bands was more than a century later in 1982 (figure 2*c*), also a strong El Niño year, but superimposed onto an anthropogenic warming trend. The next major event, in 2002, marked the onset of frequent heat stress in the twenty-first century, with multiple stress bands also present in 2010, 2015, and 2016 (figure 2). Accordingly, there was a significant increase in stress band occurrence over time since 1815 (*p* < 0.0001, mixed effects model), with some influence of reef location (significantly more stress bands in Yonge Reef and Nouméa compared to the Coral Sea) but no significant effect of depth (*p* = 0.34). Continuous direct observations since the 1970s around Lizard Island in the northern GBR generally confirm the history recorded in our cores, with the timing of stress bands matching coral bleaching reports in 1982, 2002, and 2016 [7,25]. Our cores also reveal evidence of stress responses in 2010 and 2015 (figure 2), even though DHW values were moderate (less than 4°C-weeks). However, the proportion of cores with stress bands decreases between 2015 and 2016, with no stress bands observed in 2017, even though 2016 and 2017 were the years with highest DHW (figure 1).

Although early studies of stress bands in *Orbicella* (formerly *Montastrea*) corals from the western Atlantic Ocean suggested that stress bands may form under exposure to either anomalously warm or cold water [13,14], more recent studies with Indo-Pacific *Porites* have consistently linked stress bands to high-temperature events [16–19,23]. Indeed, our stress band analysis successfully captured known bleaching events in the northern GBR and Coral Sea during 1982 and 2002 [25,26]. Yet, widespread bleaching of corals, including *Porites*, was also reported across the northern GBR and the Coral Sea in 2016 and 2017 [7,8,27], and half of the cored colonies in Nouméa bleached in 2016. The presence of only a few stress bands in 2016 and none in 2017 is thus the first identified decoupling between coral bleaching and the occurrence of stress bands in *Porites* skeletal cores. When corals lose the symbionts that provide most of their energy, they begin to consume their own tissues (i.e. catabolysis), eventually dying if bleaching persists for long enough that their tissue reserves are depleted [28]. Stress bands form as a result of the cessation of upward growth and depletion of the tissue layer during catabolysis [18]. Therefore, the absence of stress bands and continued skeletal accretion during summer heatwaves in 2016–2017 suggests that the corals may have nourished themselves with heterotrophic feeding [29], stored energy in expanded tissue reserves prior to bleaching, or diverted resources from other physiological processes such as reproduction [28]. The increase in tolerance to repeated heat stress is also consistent with recent suggestions that coral bleaching may be viewed as an immune system response with ‘memory’ of previous exposures [30]. Whichever mechanism was in place, our results indicate that the observed tolerance to high DHW in 2016–2017 was established only after 2015 because higher proportions of stress bands were found in previous years (figure 2).

There are at least two alternative explanations for the complete absence of 2017 stress bands. The first is that our sampling occurred too early after the 2017 bleaching event for the corals to accrete clearly visible stress bands. However, our cores were collected six to nine months after peak temperatures in 2017, and a previous study found that this is sufficient time for the corals to continue growing above any newly formed stress bands such that they are visible in CT scans [17]. Thus, the lack of 2017 stress bands is unlikely to be an artefact of the time of sampling. Alternatively, it is possible that other environmental factors, such as localized currents or cloud cover, dampened heat stress for these corals in 2017. Indeed, the occurrence of stress bands in non-heatwave years, albeit in small proportions of our cores, supports the notion that local environmental factors can influence stress, and thus potentially stress band presence or absence. Nevertheless, that we found high proportions of stress bands in previous bleaching years, combined with the success of *Porites* stress bands in recording bleaching events in several other studies [16–19,23], suggest that the absence of 2017 stress bands is unlikely a result of local environmental variability across our broad study region, but rather it is more likely due to prior exposure to heat stress events in 2015 and 2016.

**Figure 3.**
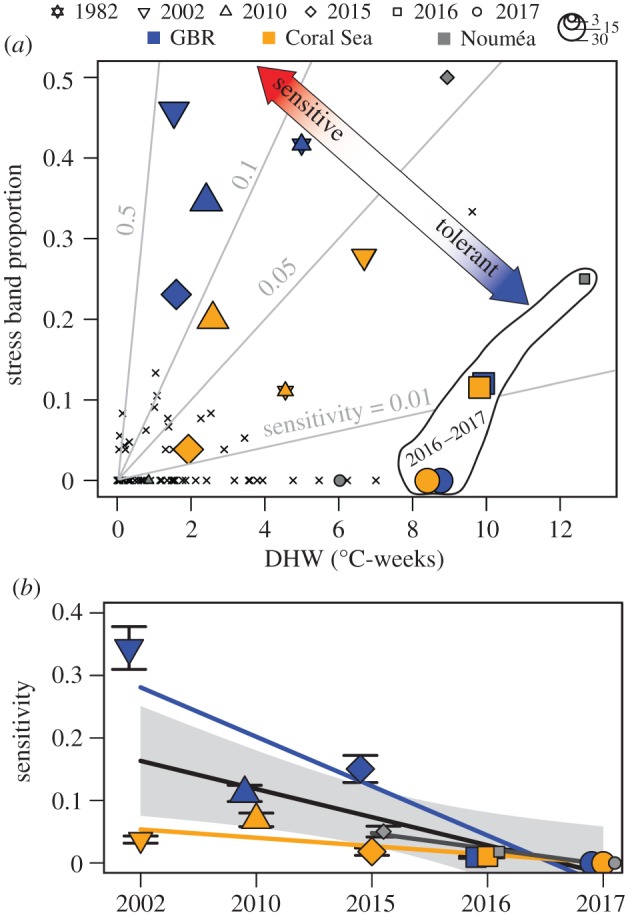
Declining sensitivity to heat stress. (*a*) Proportion of cores with stress bands in all years of the satellite era (1982–2017, crosses) and the years with the most evidence of heat stress (symbols indicate year, colours indicate location, and symbol size indicates number of replicates). Light grey lines show constant sensitivities to heat stress, defined as the ratio of stress band proportion to maximum DHW. (*b*) Sensitivity to heat stress in the twenty-first century (colours are the same as *a*). Regression lines are shown for each region (blue, grey, and orange) and for the pooled data (black), with the grey error bound indicating the 95% confidence interval of the pooled fit. Error bars indicate uncertainty (1 *σ*) based on a 10% rate of false positives in stress band assignments.

Throughout the satellite SST era (1982–present), the relationship between stress bands and DHW over time follows neither a line of constant sensitivity (grey lines in figure 3*a*) nor a threshold response above a certain DHW value, as is assumed in projections of coral reef futures [5,6]. Instead, the data cluster around high-stress band proportion under low-DHW in 2002, 2010, and 2015; and low-stress band proportion despite high DHW in 2016–2017 (figure 3*a*). Even though there is a positive correlation between DHW and stress band proportion spatially across sites in 2016–2017, the relationship between DHW and stress bands (i.e. sensitivity) changed drastically among years, with a significant decline in sensitivity over time when data from all reefs are pooled (*p* = 0.013, simple linear model) or treated separately for the Coral Sea (*p* = 0.049) and GBR (*p* = 0.017). Sensitivity declined most significantly on the GBR, mainly due to the higher proportions of stress bands there in 2002 and 2010. Sensitivity either remained stable or slightly decreased between 2002 and 2015, followed by a sharp decline in 2016 and 2017 (figure 3*b*). Similarly, there is a significant negative correlation (*p* = 0.007, mixed effects model) between DHW and stress band occurrence when considering only the anomalously warm years of 2002, 2010, and 2015–2017. Evidence for acclimatization may also be found in the timeline of stress bands within individual cores. Corals with 2002 stress bands were more likely to have 2010 stress bands (odds ratio 1.95), and those with 2010 stress bands were more likely to have 2015 stress bands (odds ratio 1.71). By contrast, corals with a stress band in 2015 were less likely to have one in 2016 (odds ratio 0.64), and no corals had stress bands in 2017. Thus, our data suggest that sensitivity to DHW remains stable when the return time of heat stress exceeds several years (e.g. 2002, 2010, and 2015), but corals can develop tolerance (i.e. decreased sensitivity) to heat stress in response to more frequent heatwaves (e.g. 2015, 2016, and 2017). Whereas a previous study inferred that acclimatization mechanisms will be quickly overridden by global warming [31], our findings indicate that the temperature tolerance of *Porites* has increased as the return time of heat stress events approaches 1 or 2 years.

While our results demonstrate that massive *Porites* corals increased their thermal tolerance during 2016–2017 in response to modest heat stress in 2015, it is possible that similar pre-conditioning responses have been expressed before, and potentially by other coral species. In the broader Coral Sea and northern GBR region, heat stress in 2004 exceeded that of 2002 in most areas, and even rivalled that of 2016/2017 at some reefs (figure 1; electronic supplementary material, figure S1). While both mass coral bleaching [26] and stress band formation (figure 2) occurred in 2002, there are neither reports of bleaching [26] nor a high proportion of stress bands (only 2/24) during 2004 in the northern GBR. Thus, it is conceivable that increased thermal tolerance after the modest 2002 warming event mitigated corals from bleaching in 2004, a notion that is consistent with reports of both species- and community-level acclimatization in other field and laboratory studies [4,10–12,31–33]. Conversely, record-breaking temperatures in 2016 and 2017 together killed approximately half of the corals on the GBR [8], even though they were exposed to modest warming in 2015. This can be interpreted in several ways. First, the concurrence of a strong El Niño with peak summer in 2016 may have created such an extreme heatwave [34] that many corals succumbed irrespective of their ability to augment their thermal tolerance. Alternatively, exposure to modest warming in 2015 may have diminished the effects of heat stress on the survivors during the following 2 years, leaving populations that are better able to withstand recurring heatwaves [8,35]. Finally, because many massive-morphology corals, including *Porites*, exhibit characteristics of a stress-tolerant life-history strategy [36], they may have greater ability to survive heat stress than corals with weedy or competitive life-history strategies [8,36].

As the rate of severe heat stress events increases [26,37], adaptation and acclimatization in reef-building corals is essential to mitigate against rapid environmental change [8,35] and preserve ecosystem services [26,38]. The capacity of the hundreds of coral species that inhabit reefs to increase their thermal tolerance in the face of global warming will determine whether coral reefs' rich biodiversity [39] will persist into the next century, or whether future generations will inherit reefs covered by depauperate coral communities that would be unrecognizable to us today as functioning coral reef ecosystems. Our findings indicate that natural mechanisms to tolerate the increased frequency of marine heatwaves have been triggered and provide a glimmer of hope that at least some corals can acclimatize to repeated heat stress, enabling them to persist in a warmer world.

## Supplementary Material

Supplementary Material

## Data accessibility

Datasets supporting this article have been uploaded as electronic supplementary material, and the raw data and code can be found online at https://codeocean.com/capsule/9809895/tree/v1 (https://doi.org/10.24433/CO.7136833.v1).
