# Supplementary material for: Acclimatization of massive reef-building corals to consecutive heatwaves
Source: Proc Biol Sci. 2019 Mar 6;286(1898):20190235. doi: 10.1098/rspb.2019.0235 (PMC6458333; doi:10.1098/rspb.2019.0235)
Supplement: Supplementary Material [file rspb20190235supp1.pdf]

Supplementary Information for:

Acclimatization of massive reef-building corals to consecutive heatwaves

Thomas M. DeCarlo*, Hugo B. Harrison, Laura Gajdzik, Diego Alaguarda, Riccardo Rodolfo-Metalpa, Juan D'Olivo, Gang Liu, Diana Patalwala, and Malcolm T. McCulloch

*Correspondence to: thomas.decarlo@uwa.edu.au

Supplementary Figures S1-S5

Supplementary Tables S1-S5

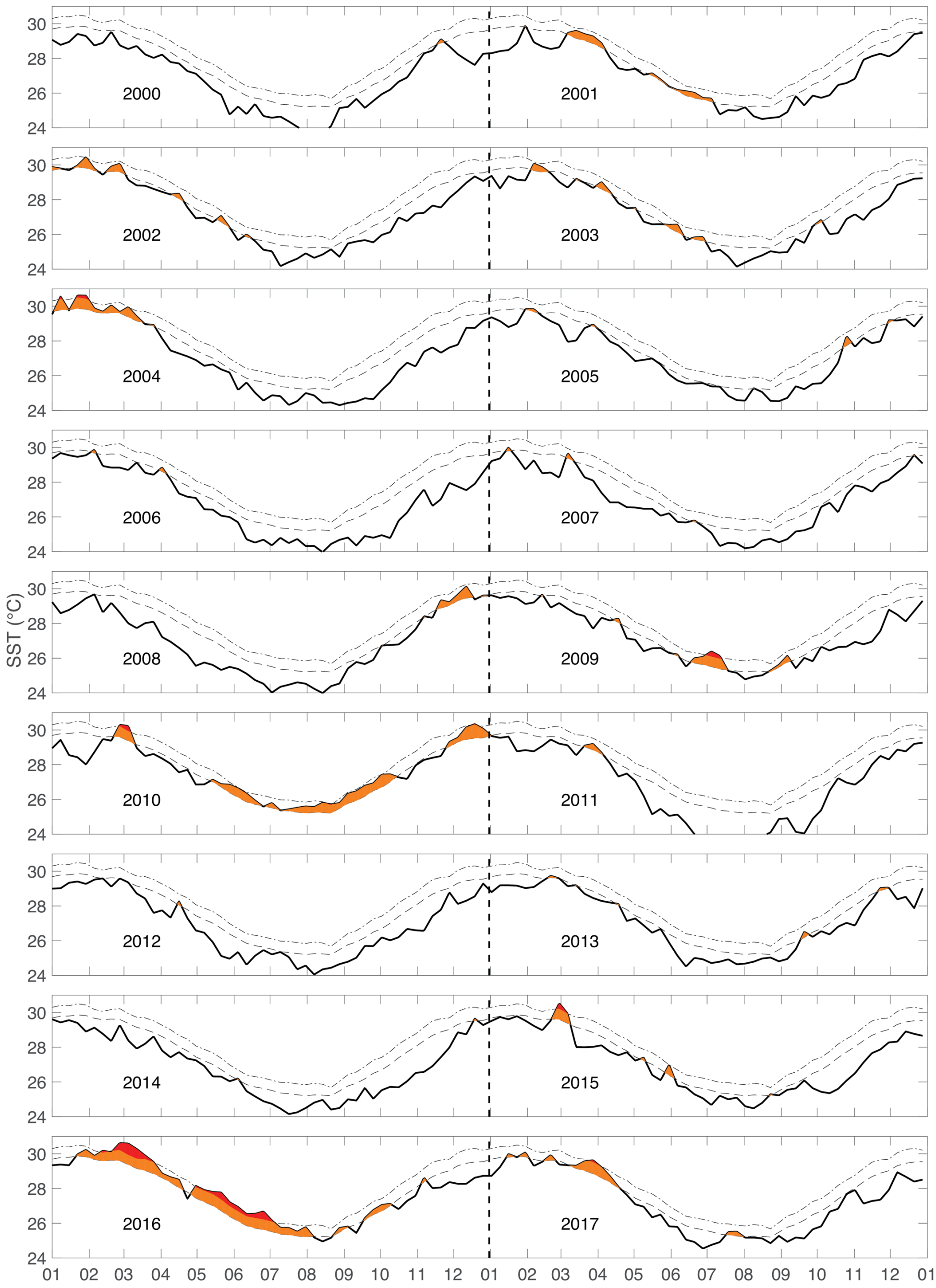

Supplementary Figure S1. Marine heatwaves on the northern GBR in the 21st century. Solid black line shows the weekly NOAA Optimum interpolation SST (1° spatial resolution) data for our northern GBR sites from 2000-2017. The dashed and dot-dashed lines show thresholds for Category 1 (orange) and Category II (red) marine heat waves [37]. The x-axis shows months of the year. Summertime Category II heatwaves were reached in 2004, 2010, 2015, 2016, and 2017.

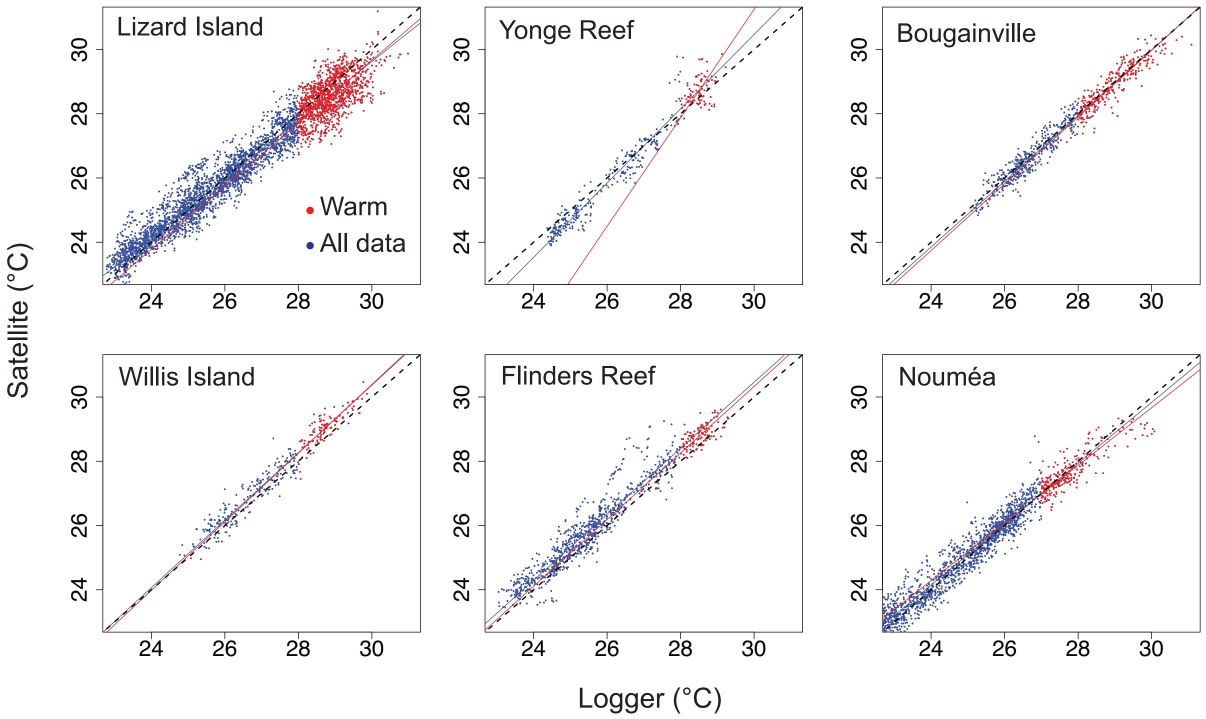

Supplementary Figure S2. Validation of satellite SST with *in-situ* loggers. Each panel compares daily mean SST from *in situ* temperature loggers [21,22] (x-axis) with NOAA CRW satellite SST (y-axis). Blue and red colours indicate all and warm (>28 °C, except for Nouméa >27 °C) time points, respectively. Regression lines show reduced major axis (RMA) fits to all data (blue) and warm data (red), and dashed black lines show 1:1 relationships.

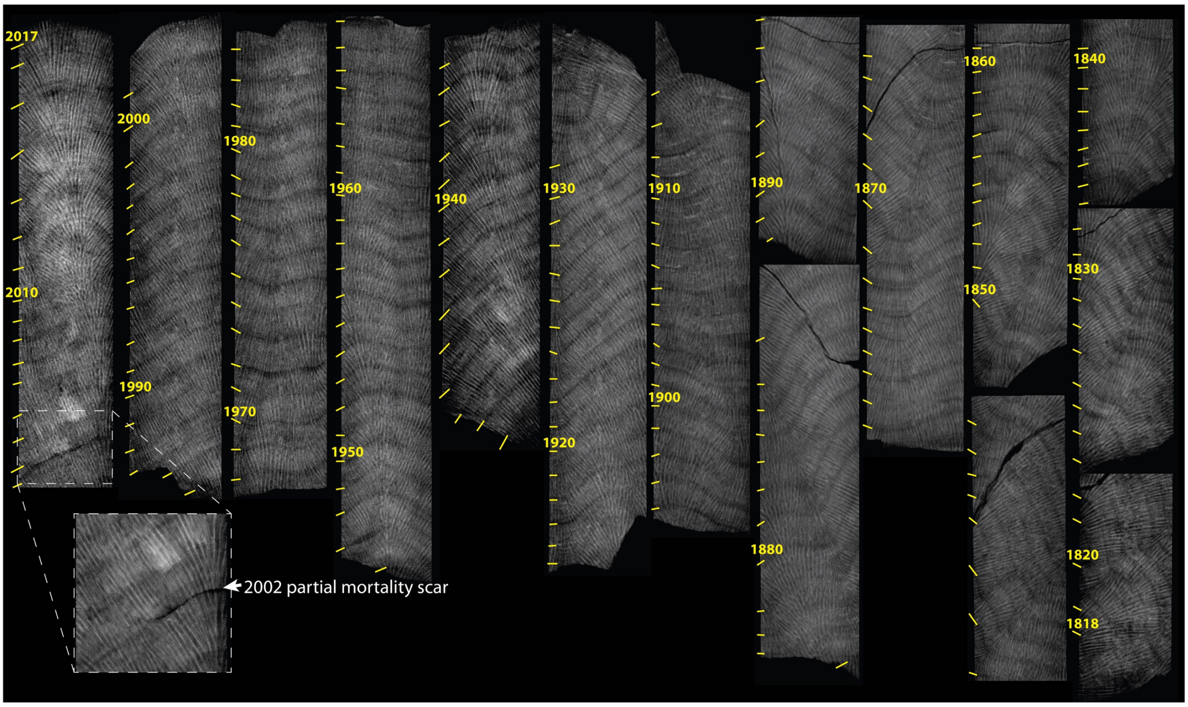

Supplementary Figure S3. Compiling coral core CT scans to develop centuries-long chronologies. Multiple μCT scans (dark/light shading = low/high density) of pieces of the same core are connected to develop a full chronology for core CS19. Yellow tick marks indicate annual low-density bands, with each decade is labelled in yellow text. The first evidence of heat stress is a partial mortality scar dated to 2002 (inset image). Note that the inset image was acquired after slight 3D rotation and tilting to obtain the clearest view of the partial mortality scar.

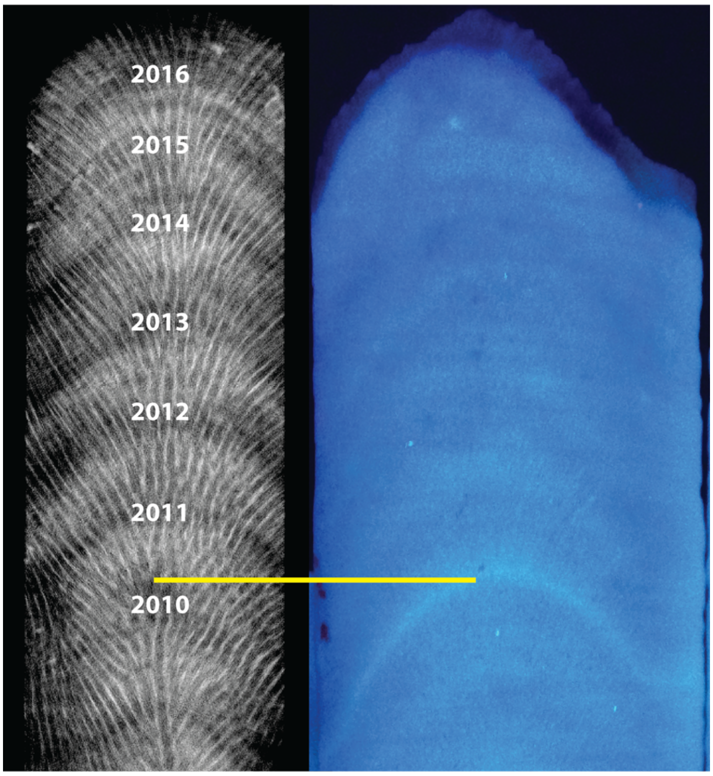

Supplementary Figure S4. Validation of µCT chronologies with luminescence banding visible under UV light. A µCT scan (left) of core LIZ17-01 with clear density banding is matched to the bright luminescence band (right) visible in the same core, indicating that the bright luminescence band formed between 2010 and 2011. The year labels on the µCT image are placed on the low-density band, with the partial development of a high/low density band pair above 2016 interpreted as 2017 since the core was collected in early October 2017.

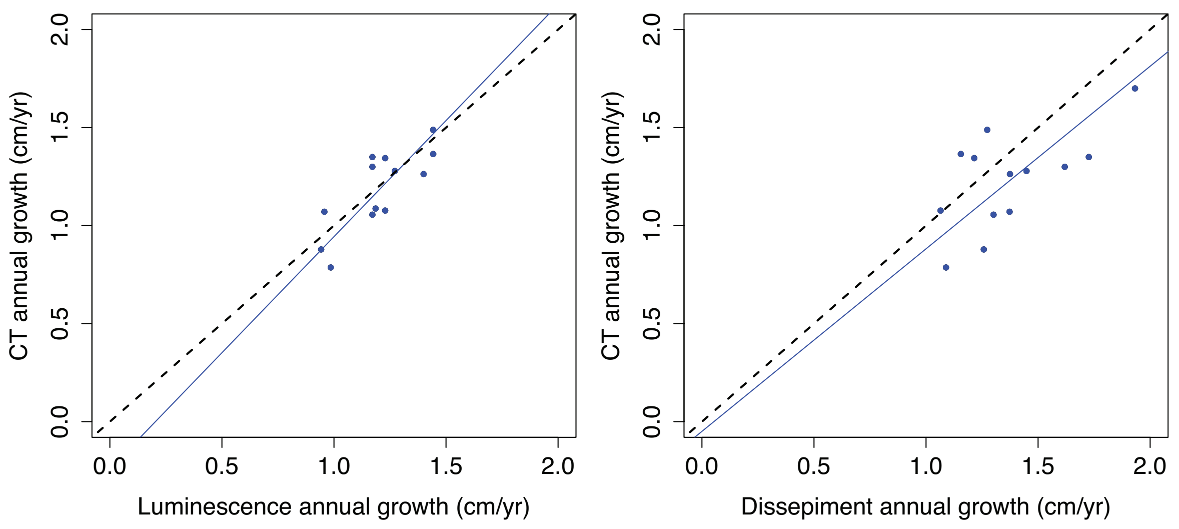

Supplementary Figure. S5. Validation of coral chronologies by comparing linear extension estimates from μCT, luminescent bands, and dissepiment spacing. Each point represents the comparison of annual extension rate measured via two techniques on the same colony, solid blue line indicates RMA regression, and dashed black line indicates a 1:1 relationship.

Supplementary Table S1. Satellite-logger calibration slopes. “Warm” is defined as >28 °C for all locations except Nouméa, where it is >27 °C. Slopes were derived from reduced major axis (RMA) regressions. CI = “confidence interval”.

Site	RMA slope	RMA slope 95% CI	RMA slope warm	RMA slope warm 95% CI
Lizard Island	0.91	0.90-0.92	0.98	0.91-1.06
Yonge Reef	1.16	1.13-1.18	1.69	1.01-2.84
Bougainville Reef	1.02	1.01-1.04	1.04	0.98-1.10
Willis Island	1.05	1.03-1.08	1.07	0.94-1.20
Flinders Reef	1.03	1.01-1.04	1.04	0.85-1.26
Nouméa	0.95	0.94-0.96	0.90	0.84-0.96

Supplementary Table S2. Northern Great Barrier Reef coral coring locations, depths, colony ages (earliest dated growth band), and stress band years.

Core ID	Reef location	Latitude	Longitude	Depth (m)	Age	Stress band years
LIZ17-01	Lizard Island	-14.68683	145.44128	2	1912	1968
LIZ17-02	Lizard Island	-14.68683	145.44128	4	1994	2010
LIZ17-03	Lizard Island	-14.68683	145.44128	4	1999	2002, 2015
LIZ17-04	Lizard Island	-14.68756	145.46576	7	1916	1982
LIZ17-05	Lizard Island	-14.68726	145.46509	2.5	1935	1976
LIZ17-06	Lizard Island	-14.68726	145.46509	2.5	1972	1982, 1995, 2015
LIZ17-07	Lizard Island	-14.68934	145.46725	2.5	2002	2002, 2006, 2010, 2016
LIZ17-08	Lizard Island	-14.68933	145.46748	2.5	1932	1998, 2002, 2011
LIZ17-09	Lizard Island	-14.68577	145.44197	1	1993	2010, 2015
LIZ17-10	Lizard Island	-14.68577	145.44197	1	1990	2002, 2010
YON17-01	Yonge Reef	-14.57188	145.61493	9.5	1902	1910, 1949, 1982, 1998, 2015
YON17-02	Yonge Reef	-14.57803	145.61040	7.5	1969	2004, 2010, 2016
YON17-03	Yonge Reef	-14.57803	145.61040	8	1939	1995, 2002
YON17-04	Yonge Reef	-14.57078	145.61623	3	1855	1877, 2011, 2016
YON17-05	Yonge Reef	-14.57624	145.61122	7.5	2008	N/A
YON17-06	Yonge Reef	-14.57624	145.61122	8	1970	1982, 2015
YON17-07	Yonge Reef	-14.57624	145.61122	8	1976	1982, 1994, 2002, 2009
YON17-08	Yonge Reef	-14.57624	145.61122	7	1989	1989, 2002, 2010, 2012
YON17-09	Yonge Reef	-14.57624	145.61122	7	1991	2002, 2010
YON17-10	Yonge Reef	-14.57624	145.61122	9	1988	1990, 2002, 2010, 2015
MAC17-01	MacGillivray Reef	-14.64767	145.48752	5.5	1986	2002, 2004, 2014
MAC17-02	MacGillivray Reef	-14.64767	145.48752	5	1997	2002
MAC17-03	MacGillivray Reef	-14.64767	145.48752	6.5	1997	N/A
MAC17-04	MacGillivray Reef	-14.64767	145.48752	5	2008	N/A
MAC17-05	MacGillivray Reef	-14.64767	145.48752	5	1998	2001, 2006, 2013
MAC17-06	MacGillivray Reef	-14.65400	145.48985	3	1921	1924, 2010

Supplementary Table S3. Coral Sea coral coring locations, depths, colony ages (earliest dated growth band), and stress band years.

Core ID	Reef location	Latitude	Longitude	Depth (m)	Age	Stress band years
CS01	Bougainville	-15.48429	147.10564	1	1949	1982, 2002, 2008, 2013, 2016
CS02	Bougainville	-15.48394	147.10576	2	2000	N/A
CS03	Bougainville	-15.48378	147.10583	2	1990	N/A
CS04	Bougainville	-15.48195	147.105	4	1925	1940, 1996, 2002, 2016
CS05	Bougainville	-15.48341	147.11203	2	1979	1980, 2002
CS06	Bougainville	-15.48341	147.11203	2	2001	N/A
CS07	Bougainville	-15.51208	147.13206	9	N/A	N/A
CS08	Bougainville	-15.51208	147.13206	12	N/A	N/A
CS09	Bougainville	-15.51208	147.13206	14	2008	2010
CS10	Moore Northwest	-15.88387	149.15414	5	1986	N/A
CS11	Moore Northwest	-15.87998	149.15758	8	1981	1998
CS12	Moore Northwest	-15.87998	149.15758	8	1995	N/A
CS13	Moore Northwest	-15.89456	149.16384	16	2008	N/A
CS14	Moore Southeast	-15.96871	149.1927	2	1815	N/A
CS15	Diane Bank	-15.72173	149.61452	7	2006	2010
CS16	Diane Bank	-15.72331	149.61786	20	2006	2010
CS17	Diane Bank	-15.72095	149.61618	10	1998	N/A
CS18	Willis North Cay	-16.13444	149.97911	10	2012	N/A
CS19	Willis Middle Cay	-16.21289	149.98933	11	1818	2002, 2003
CS20	Willis South Cay	-16.29156	149.96413	8	N/A	N/A
CS21	Magdalene North	-16.52323	150.27562	14	2008	N/A
CS22	Magdalene North	-16.52425	150.27684	13	1964	N/A
CS23	Magdalene North	-16.5242	150.27827	14	1922	N/A
CS24	Magdalene South	-16.59631	150.33476	10	2006	N/A
CS25	Magdalene South	-16.59631	150.33476	10	2009	N/A
CS26	Magdalene South	-16.59955	150.32698	17	1997	1998, 2002, 2010, 2016
CS27	Magdalene South	-16.59955	150.32698	13	2001	2007, 2010, 2012, 2015
CS28	Flinders Reef	-17.70472	148.46541	4	1835	N/A
CS29	Flinders Reef	-17.70472	148.46541	5	N/A	N/A
CS30	Flinders Reef	-17.71715	148.44731	6	1990	N/A

Supplementary Table S4. Nouméa coral coring locations, depths, colony ages (earliest dated growth band), and stress band years.

Core ID	Reef location	Latitude	Longitude	Depth (m)	Age	Stress band years
NOU Z9A	Nouméa	-22.29577	166.43535	3	2012	2015
NOU Z8A	Nouméa	-22.29577	166.43535	3	2012	2015
NOU Z6A	Nouméa	-22.29577	166.43535	3	2008	N/A
NOU Z2A	Nouméa	-22.29577	166.43535	3	2012	N/A
NOU B1A	Nouméa	-22.29577	166.43535	3	2015	2015
NOU B8A	Nouméa	-22.29577	166.43535	3	2007	2015
NOU B9A	Nouméa	-22.29577	166.43535	3	2013	N/A
NOU B12A	Nouméa	-22.29577	166.43535	3	2007	2009, 2016

Supplementary Table S5. Statistical results.

Model	Slope	p value	n	factor(s)	r^2	F
Stress band occurrence vs. time (yrs)	0.030 ± 0.006	$1x10^{-6}$	2644 years<br><br>65 cores	depth (p = 0.34)<br>Noumea (p = 0.02)<br>Yonge (p = 0.006)<br><br>Cores as random factors		
Sensitivity vs. time (yrs)	-0.05 ± 0.02	0.013	13	-	0.44	$F_{1,11} = 8.74$
stress band occurrence vs. DHW	-0.13 ± 0.05	0.007	276	No sites were significant factors<br><br>Cores as random factors		

Notes: ± indicates standard error. Sensitivity vs. time was performed with simple linear regression and has r^2 and F statistics, whereas Stress band occurrence vs. time and stress band occurrence vs. DHW were tested with generalized linear mixed effects models.
